# On the Potential of a New Generation of Magnetometers for MEG: A Beamformer Simulation Study

**DOI:** 10.1371/journal.pone.0157655

**Published:** 2016-08-26

**Authors:** Elena Boto, Richard Bowtell, Peter Krüger, T. Mark Fromhold, Peter G. Morris, Sofie S. Meyer, Gareth R. Barnes, Matthew J. Brookes

**Affiliations:** 1 Sir Peter Mansfield Imaging Centre, School of Physics and Astronomy, University of Nottingham, University Park, NG7 2RD, Nottingham, United Kingdom; 2 Midlands Ultracold Atom Research Centre, School of Physics and Astronomy, University of Nottingham, University Park, NG7 2RD, Nottingham, United Kingdom; 3 Wellcome Trust Centre for Neuroimaging, University College London, 12 Queen Square, WC1N 3BG, London, United Kingdom; Australian Research Council Centre of Excellence in Cognition and its Disorders, AUSTRALIA

## Abstract

Magnetoencephalography (MEG) is a sophisticated tool which yields rich information on the spatial, spectral and temporal signatures of human brain function. Despite unique potential, MEG is limited by a low signal-to-noise ratio (SNR) which is caused by both the inherently small magnetic fields generated by the brain, and the scalp-to-sensor distance. The latter is limited in current systems due to a requirement for pickup coils to be cryogenically cooled. Recent work suggests that optically-pumped magnetometers (OPMs) might be a viable alternative to superconducting detectors for MEG measurement. They have the advantage that sensors can be brought to within ~4 mm of the scalp, thus offering increased sensitivity. Here, using simulations, we quantify the advantages of hypothetical OPM systems in terms of sensitivity, reconstruction accuracy and spatial resolution. Our results show that a multi-channel whole-head OPM system offers (on average) a fivefold improvement in sensitivity for an adult brain, as well as clear improvements in reconstruction accuracy and spatial resolution. However, we also show that such improvements depend critically on accurate forward models; indeed, the reconstruction accuracy of our simulated OPM system only outperformed that of a simulated superconducting system in cases where forward field error was less than 5%. Overall, our results imply that the realisation of a viable whole-head multi-channel OPM system could generate a step change in the utility of MEG as a means to assess brain electrophysiological activity in health and disease. However in practice, this will require both improved hardware and modelling algorithms.

## Introduction

Magnetoencephalography (MEG; [1, 2]) is a non-invasive technique to image electrophysiology in the human brain. It is based on assessing the changes in magnetic field outside the head that are generated by synchronised current flow through neuronal assemblies in the brain. These extra-cranial magnetic field fluctuations are of order ~10^−14^ T and are typically detected via an array of superconducting coils placed around the head, each coupled to a superconducting quantum interference device (SQUID) [3, 4]. Appropriate mathematical modelling of the measured magnetic field facilitates reconstruction of 3-dimensional current density images, which depict moment-to-moment changes in the brain’s electrical activity. Recent years have seen rapid improvement in the utility of MEG, driven primarily by improved algorithms for data analysis. The MEG inverse problem (generating 3D current density images using measurements of extra-cranial magnetic fields) is ill-posed (i.e. no unique solution exists), which makes the direct inference of spatial dynamics mathematically complex. However, many algorithms (for example minimum-norm [5, 6] and beamforming [7–11]) now exist to verifiably reconstruct the spatial, temporal and spectral signature of human brain activity with reasonable (~5–10 mm; [12, 13]) spatial resolution and excellent (<1 ms) temporal resolution. This capability makes MEG one of the most exciting tools for basic neuroscientific and clinical research that is currently available. Multiple studies [14, 15] have shown that appropriately modelled MEG signals are rich in information content and strikingly similar to invasive electrophysiological measurements made in animals and humans. Further, MEG studies have had significant impact on our understanding of neural networks [16–22], allowing elucidation of intrinsic modes of electrophysiological coupling between spatially separate and functionally specific brain regions [23]. Most importantly, MEG is providing new information on the pathophysiology of a range of diseases and conditions including developmental disorders [24], psychoses [25] and neurodegeneration [26].

Despite its excellent and unique potential, MEG is limited by a low signal-to-noise ratio (SNR) and restricted spatial resolution. Magnetic fields generated by the brain are much smaller in magnitude than environmental and biomagnetic interference. This problem is observed directly in comparisons of MEG with invasive electrophysiology; for example, comparing MEG measurements of Zumer et al. [15] to invasive measurements by Mukamel et al. [27], despite good overall agreement, the sensitivity of MEG at high frequency (i.e. measuring neural oscillations above ~50 Hz) decreases due to poor SNR. In principle, SNR could be increased by moving detectors closer to the head (the field from dipoles in the brain follows an inverse square law, meaning that halving the brain to detector separation would quadruple SNR). However, in SQUID-based systems, the requirement for cryogenic cooling of pickup coils and SQUIDs means that a vacuum must be maintained between the head and the coils, thus limiting the minimum separation. Spatial resolution in MEG is governed by our ability to separate magnetic fields generated by sources in spatially separate brain locations; this is affected by the inverse modelling algorithm, but also by sensor coverage, sensor type and SNR. In general, poor spatial resolution means that reconstructed signals at separate locations are spuriously correlated, particularly for locations in close proximity (i.e. nearby sources become linearly mixed). This impacts directly on our ability to adequately characterise brain networks; for example in the study of connectivity (measuring statistical interdependencies between regions; [23, 28, 29]) methodologies [20, 30–33] have to be used for post-hoc correction to reduce such spurious correlations, and the spatial scale of the networks that can be assessed is limited. If these restrictions on SNR and spatial resolution could be mitigated, this would afford a step change in the utility of MEG as a functional neuroimaging modality.

Optically-pumped magnetometers (OPMs) measure the transmission of laser light through a vapour of spin-polarised rubidium atoms, which provides a highly sensitive measure of the local magnetic field. These devices are emerging as an alternative to SQUIDs for measuring small magnetic fields and recent studies have begun to show their viability for a number of biomagnetic applications, including MEG. OPMs with sensitivities surpassing SQUIDs’ have been demonstrated [34, 35]; working in the spin-exchange relaxation-free (SERF) regime [36, 37]. Several papers have described the successful OPM detection of phase-locked evoked responses generated by auditory [38–40] or somatosensory [38, 41] stimulation. Other studies have shown that neural oscillations (rhythmic electrical activity in neural networks) can also be detected [41, 42]. In addition, the detection of epileptiform activity in rodents using micro-fabricated OPM sensors [43] has been shown. These empirical demonstrations, coupled with miniaturization of OPM cells to the millimetre scale [44] and the introduction of the first multi-channel measurements [45, 46] have begun to suggest that OPM technology could transform the utility of MEG with chip-scale sensors, in principle, facilitating the use of several hundred detectors, located just millimetres from the scalp surface, with a completely flexible geometry.

Qualitatively the advantages afforded by an OPM-based MEG system are obvious—bringing sensors closer to the head will increase the measured signal; assuming noise floors of OPMs and SQUIDs are comparable, this would lead to increased SNR in MEG measurements. Although this result is expected, many critical questions remain unanswered: What *quantitative* improvement in SNR might be expected and how does this change with cortical location? How would this SNR gain affect source reconstruction accuracy (following inverse modelling)? How would an OPM system be impacted by the accuracy of modelling algorithms? What would the gain in spatial resolution be compared to current systems? In addition, the number of sensors required remains an open question. In general it is well known that increased sensor density improves spatial resolution. However, at high SNR spatial resolution has been shown to be asymptotic [47] with respect to the number of sensors. Given the potential for high SNR when using OPM systems, this potentially means that beyond some critical number, the addition of further sensors may not generate significant improvement in spatial specificity. In the present paper, we quantify the potential advantages of several theoretical MEG systems in which sensors are placed directly on the scalp surface. Note that in principle our theoretical systems could result from any technology in which the external surface of the magnetic field sensor is at room temperature, for example nitrogen-vacancy centres in diamond [48]; however we specifically use the term OPM throughout the paper due to the excellent potential of these devices both in terms of achieving close detector proximity to the head and their size (millimetre scale OPMs mean systems with many hundreds of sensors are plausible). Note also that our paper does not address the issues relating to the practical use of OPMs, rather our aim is to provide a benchmark to which any real system might aspire.

Using simulations, we investigate the MEG forward field in order to quantify the improvement in sensitivity of an OPM system compared to a current state-of-the-art SQUID system. Following this, we employ a beamformer spatial filtering approach to quantify the advantages of an OPM system for source reconstruction. We examine reconstruction accuracy of beamforming for single dipoles. In addition, by combining dipolar sources of interest with interference generators, we examine the efficacy of interference rejection in OPM and SQUID systems. We investigate how the accuracy of magnetic field modelling (i.e. the accuracy of the modelled forward field) impacts the OPM and SQUID systems. Finally, we examine the spatial resolution of OPM and SQUID systems, its reliance on the number of channels, and how this might impact connectivity metrics. Our results show that OPM systems, built to the geometries that we suggest, have the potential to offer a step change in the utility of MEG, with significant improvements in sensitivity and spatial resolution well beyond what could ever be expected of current instruments. However, we also show that the advantages of these next generation systems are critically dependent on our ability to generate accurate forward models, meaning that realisation of the true benefits will only result from concerted efforts in both hardware development and mathematical modelling.

## Methods

### Simulated systems

#### sSQUID

For all simulated SQUID-based measures, we employed the third-order, synthetic gradiometer configuration of a 275-channel CTF whole-head MEG system (MISL, Canada). This system contains 275 radially-oriented, axial gradiometers with a 5 cm baseline, along with a 29-channel reference array that is used to construct third-order synthetic gradiometers. The position of the head within the MEG helmet was based on an experimental recording, and was determined by using three head position indicator coils attached to the subject’s head (nasion, left pre-auricular and right pre-auricular fiducial markers). In this system, the minimum distance from the scalp surface to the closest detector coils is 1.7 cm; this would assume that the scalp is touching the surface of the MEG helmet (which of course is not simultaneously achievable for all scalp locations). In the simulations used here, the subject was positioned with the back of their head touching the back of the helmet, meaning that the largest scalp-to-sensor distance was at the front of the head for the sSQUID system. High resolution anatomical magnetic resonance images (1 mm^3^ resolution), including fiducial markers, were available for this subject allowing complete registration of the MEG sensor geometry to the brain anatomy.

#### sOPM

In the simulated OPM system, measurement locations were 4 mm from the scalp surface; a distance which is realistic for currently available SERF OPMs [49]. The arrangement of sensors was determined by the projection of the 275 sensor locations from the sSQUID system onto the scalp of the same individual used for the sSQUID simulations. Given the size of current micro-fabricated OPM devices (~1 cm^2^ footprint) such a 275-channel system is conceivable. The scalp surface was extracted from the anatomical MRI using Iso2Mesh (http://iso2mesh.sourceforge.net/; [50]). To ensure equivalence of the sSQUID and sOPM systems for sensitivity comparison, the sOPM system was also configured with its sensors forming radially-oriented axial gradiometers (5 cm baseline). Note however that for beamformer measurements (see later) we consider OPM magnetometers.

In both the sOPM and sSQUID simulations, simulated dipoles were located on the cortical surface, which was extracted from the anatomical MRI using Freesurfer (http://surfer.nmr.mgh.harvard.edu/; [51]). This meant that 21,401 individual dipoles could be located at the vertices of the triangular cortical mesh, with each dipole oriented perpendicular to the cortical surface. In order to simulate magnetic fields at the individual sensor locations, a multiple local sphere head model was used [52] in conjunction with the forward model derived by Sarvas [53]. Gradiometers were formed via a subtraction of the radial field components through each gradiometer loop (in the case of the sSQUID) or through two OPMs in the case of sOPM. Third-order gradiometers for the sSQUID were constructed using weighting coefficients from the MEG recording.

### Comparison of SNR in sOPM and sSQUID systems

Assuming an equivalent noise floor (≲10 fT rms/Hz^1/2^) for the sSQUID [54] and the sOPM systems (both in magnetometer [36] and gradiometer configurations), the difference in field magnitudes at the sensors can be used to calculate the gain in SNR afforded by the sOPM system (thus, in the remainder of this manuscript, all SNR calculations assume equivalent noise floors for the sSQUID and sOPM). Measured fields at each sensor were simulated for each possible dipole location in the cortex, assuming a dipole moment, │**Q**│ = 1 nAm. For every cortical location, j, we calculated the expected output (i.e. the forward field) **l**_j_ for all 275 MEG channels for both systems (therefore **l**_j_ has dimensions of N_ch_ × 1, being N_ch_ the number of channels). The Frobenius norm, f_j_, of the forward field vector **l**_j_ was then computed as,

$${\text{f}}_{\text{j}}={\left|l_{\text{j}}\right|}_{\text{F}}=\sqrt{\Sigma_{\text{j}=1}^{{\text{N}}_{\text{ch}}}{\left|{\text{l}}_{\text{j}}\right|}^{2}}$$ (1)

and the comparison of the sSQUID and sOPM systems was derived as,

$${\text{ratio}}_{\text{j}}=\frac{{\text{f}}_{\text{j},\text{OPM}}}{{\text{f}}_{\text{j},\text{SQ}}}$$ (2)

This ratio, which effectively represents the change in measured signal (and SNR) afforded by moving from the sSQUID to the sOPM system, was calculated at each source location on the cortical surface.

### Reconstruction accuracy for single dipoles

To examine the accuracy of source reconstruction, we first simulated a number of sets of MEG data. A source time course **q**_j_, at cortical location j, containing a total of N time points was simulated as Gaussian-distributed random data with a standard deviation of 5 nAm. Note here that N = f∙Δ, where f is sampling frequency and Δ represents the duration of the simulated recording. This dipole signal was projected through the forward field vector for location j, **l**_j_, and simulated MEG data were generated as,

$$m\left(\text{t}\right)=l_{\text{j}}q_{\text{j}}+e,$$ (3)

with dimensions of N_ch_ × N. Here, **e** represents noise which, for the purposes of this paper, was generated as normally-distributed random data with a standard deviation of 50 fT. A single set of MEG data, Δ = 600 s (N = 360,000) in duration, was simulated for each of the 21,401 possible dipole locations on the cortical mesh. This was done twice using forward fields computed for the sSQUID and sOPM systems. The SNR of these data was dependent on the cortical location, and varied from 0 to 3 for the sSQUID system and 0 to 24 for the sOPM system (note here SNR is computed as the standard deviation of the signal at the maximally affected channel divided by the mean noise). The difference in SNR between systems was purely a result of the proximity of the sensors to the scalp.

To compare the accuracy of source reconstruction in the two systems, a beamformer spatial filter was implemented. Beamforming [7, 9, 11, 55, 56] is a popular solution to the MEG inverse problem that has been used extensively for MEG, particularly in the study of neural oscillations and functional connectivity. An estimate of electrical source strength, **q̂**_j_, at cortical location j was given by a weighted sum of sensor measurements so that:

$${\hat{q}}_{\text{j}}=w_{\text{j}}^{\text{T}}m,$$ (4)

Note that we use the ‘hat’ notation to represent a beamformer estimate; i.e. **q̂**_j_ is the beamformer estimate of **q**_j_ made using only the MEG data, **m**. **w**_j_ is a vector of weighting parameters (dimension N_ch_ × 1) tuned to location j and derived based on variance minimisation; the overall variance in **q̂**_j_ is minimised with the linear constraint that signals originating from j remain. Mathematically,

$${\text{min}}_{{\text{w}}_{\text{j}}}\left|\langle {\hat{q}}_{\text{j}}^{2}\rangle \right|\text{ subject to }w_{\text{j}}^{\text{T}}l_{\text{j}}=1.$$ (5)

The most commonly employed solution to this equation is:

$$w_{j}^{\text{T}}=\frac{l_{j}^{\text{T}}{\left\{C+\mu \Sigma \right\}}^{-1}}{l_{j}^{\text{T}}{\left\{C+\mu \Sigma \right\}}^{-1}l_{j}}$$ (6)

where **C** = <**m**^T^ **m**> and is approximated by the data covariance. **Σ** is a diagonal matrix representing the noise at each of the MEG channels and μ is a regularisation parameter which for the purposes of this paper was set to 0.001 in order to maximise spatial resolution. Beamforming was applied in this way for all of the 21,401 simulated MEG datasets. In order to determine temporal reconstruction accuracy, the Pearson correlation coefficient was measured between the original simulated dipole time course data, **q**_j_, and the beamformer-reconstructed time course data **q̂**_j_. These correlation coefficients were computed for the sSQUID and sOPM systems and are denoted as r_SQ_(**q**_j_,**q̂**_j_) and r_OPM_(**q**_j_,**q̂**_j_), respectively.

To examine the spatial performance of the beamformer, 4 dipoles were chosen at random in the frontal, parietal and occipital regions. MEG data were generated separately for each dipole and beamformer-reconstructed time courses were generated at the source location and its 44 nearest neighbours, corresponding to distances of between 0 and 3 cm from the simulated source. The magnitude of temporal correlation between the simulated source time course and the beamformer reconstructions was then plotted as a function of distance from the source.

### Reconstruction accuracy with ‘brain noise’

**Fig 1:**
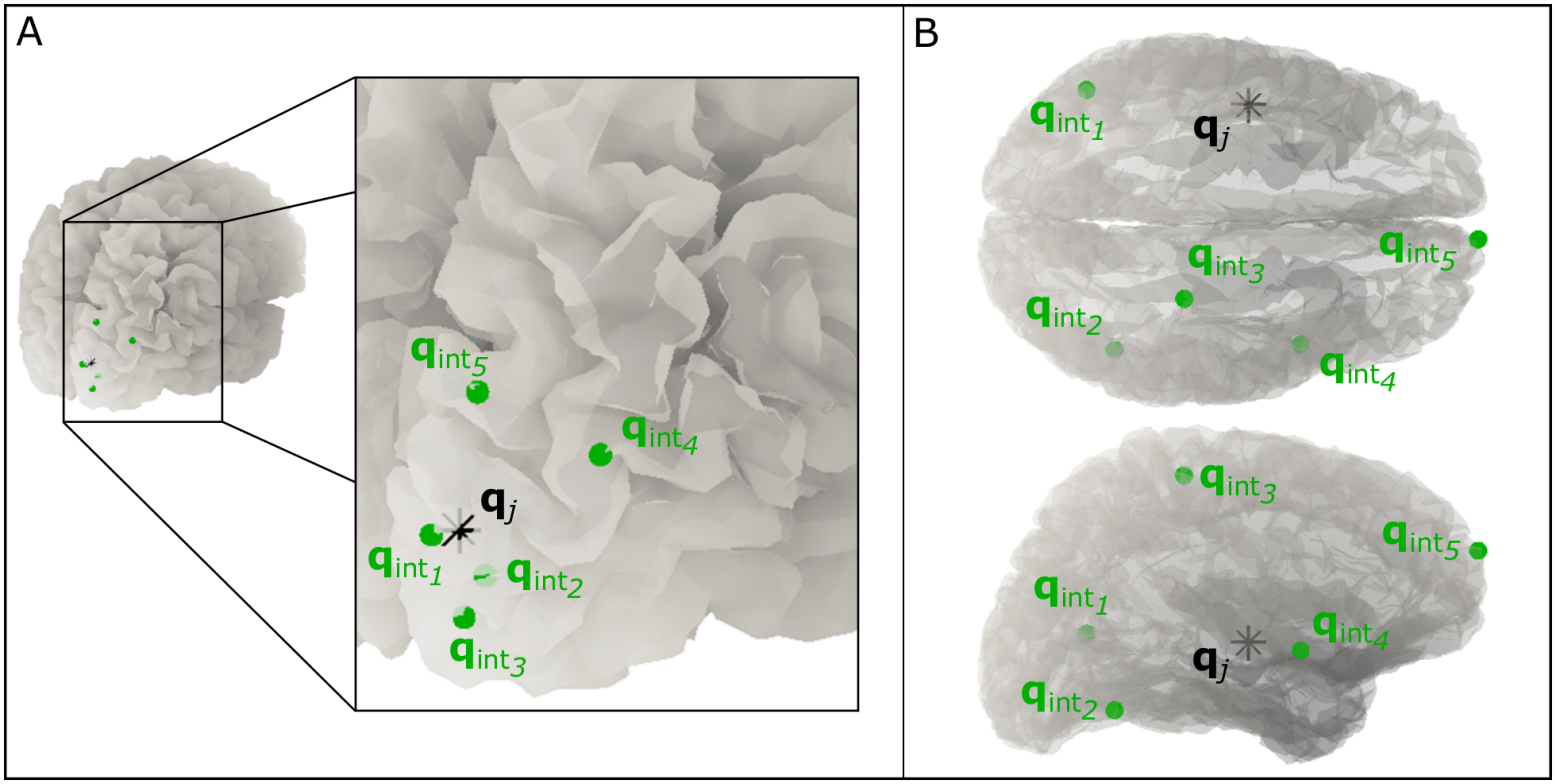
“Brain noise” sources. Schematic showing a single example of the relative locations of the source of interest (black) and the interference generators (green). (A) Case 1 (Interference in close proximity) and (B) Case 2 (Deep sources).

Although highly illustrative, beamformer reconstruction of single dipoles, as described in the previous section, is not necessarily representative of real life situations in which the beamformer must reconstruct the source of interest whilst simultaneously removing sources of interference. In order to investigate this, multiple dipole simulations were carried out. Here, a single source of interest, **q**_j_, was simulated on the cortical surface as previously. However in addition, 5 further sources, denoted by **q**_Int,k_, where k = 1, 2, …, 5, were also simulated as interference generators (brain noise). For both **q**_j_ and **q**_Int,k_, source time courses were simulated as Gaussian-distributed random data with a standard deviation of 5 nAm. The MEG data were then simulated as:

$$m\left(\text{t}\right)=l_{\text{j}}q_{\text{j}}+\left\{\Sigma_{\text{k}=1}^{5}l_{{\text{Int}}_{\text{k}}}q_{{\text{Int}}_{\text{k}}}\right\}+e$$ (7)

where **l**_Int,k_ represents the forward field vector for interference source k. Two separate cases of interference generators were simulated:

**Case 1: Interference in close proximity.** All 5 interference sources were dipolar, located on the cortical mesh, oriented perpendicular to the cortical surface and defined between 0 cm and 3 cm (Euclidean distance) from the source of interest. Actual interference locations were random, but in all cases were set such that **q**_Int,1_ was located at a distance of 0–6 mm from j; **q**_Int,2_ was between 6–12 mm from j; **q**_Int,3_ was between 12–18 mm from j; **q**_Int,4_ was between 18–24 mm from j and finally **q**_Int,5_ was between 24-mm from j. The location j, of the source of interest was allowed to vary across 10,400 possible dipole locations within a single hemisphere on the cortical mesh (see Fig 1A).
**Case 2: Deep sources.** Here, all 5 interference sources were again dipolar, oriented perpendicular to the cortical surface and located on the cortical mesh at distances less than 4 cm from the closest sOPM sensor. The source of interest, however, was limited to cortical locations between 4 cm and 6 cm from the closest sOPM sensor. This therefore simulated the situation where the source of interest was located deeper than the sources of interference. The location, j, of the source of interest was allowed to vary across 4,901 possible dipole locations (see Fig 1B).

In all cases, each MEG dataset was simulated as being 360,000 data samples (600 s) in length. In order to measure reconstruction accuracy we derived correlation coefficients r_SQ_(**q**_j_,**q̂**_j_) and r_OPM_(**q**_j_,**q̂**_j_) as previously. In order to quantify interference rejection, we also measured correlation between the beamformer-reconstructed estimate, **q̂**_j_, and all five interference source time courses; i.e. we measured r_IntSQ_(**q**_Int,k_,**q̂**_j_) and r_IntOPM_(**q**_Int,k_,**q̂**_j_) for the sSQUID and sOPM systems respectively. Obviously, for perfect beamformer performance we would expect r(**q**_j_,**q̂**_j_) to be close to one, whilst r(**q**_Int,k_,**q̂**_j_) should be close to zero.

### Reconstruction accuracy with forward field error

**Fig 2:**
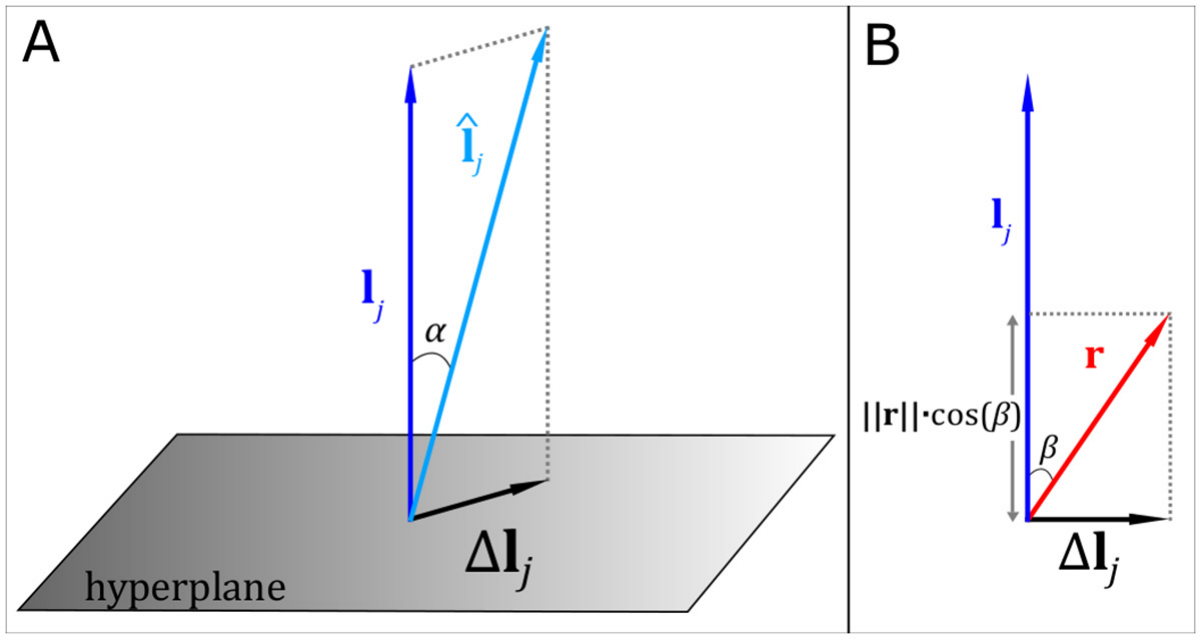
Schematic diagram of forward field error simulation. The perfect forward field, **l**_j_ is perturbed via addition of Δ**l**_j_ to give **l̂**_j_.

The simulations described in **Reconstruction accuracy for single dipoles** and **with ‘brain noise’** sections assume that the forward model used for beamforming is accurate (i.e. **l**_j_ in Eqs 3 and 7 which defines the simulated data, and **l**_j_ in Eq 6 which describes beamforming, were the same). However, in reality, some error in deriving the forward model might be expected due to, for example, inaccurate knowledge of sensor locations or poor physical models of the fields generated. For this reason, it is important to define how forward field accuracy impacts upon beamformer source reconstruction for both sSQUID and sOPM instrumentation. In order to investigate this, we first altered the beamformer weights in Eq 6 such that:

$$w_{\text{j}}^{\text{T}}=\frac{{\hat{l}}_{\text{j}}^{\text{T}}{\left\{C+\mu \Sigma \right\}}^{-1}}{{\hat{l}}_{\text{j}}^{\text{T}}{\left\{C+\mu \Sigma \right\}}^{-1}{\hat{l}}_{\text{j}}}$$ (8)

where

$${\hat{l}}_{\text{j}}=l_{\text{j}}+\Delta l_{\text{j}}$$ (9)

represents the forward field with added error Δ**l**_j_. In order to simulate error on the forward model, we employed a geometrical approach depicted in Fig 2A. We first treated the genuine forward field, **l**_j_, as a N_ch_ × 1 element vector, and defined a hyperplane in N_ch_-dimensional space that is perpendicular to **l**_j_. In order to perturb **l**_j_, we defined the error Δ**l**_j_ as a N_ch_ × 1 element vector that is confined to the hyperplane. The vector sum of **l**_j_ and Δ**l**_j_ then gave us the modified forward field as in Eq 9. The value ‖Δ**l**_j_‖/‖**l**_j_‖ represents a quantitative measure of the fractional error imposed upon the forward field model. (Note also that the angle α between **l̂**_j_ and Δ**l**_j_ (see Fig 2A) characterizes the similarity between the accurate and perturbed forward field patterns).

Δ**l**_j_ was constructed by first defining a N_ch_ × 1 vector of random numbers, **r**, as shown (for the simple 2 dimensional case) in Fig 2B. Projecting this random vector onto the hyperplane gives:

$$\Delta l_{\text{j}}=\xi \left\{r-\left(\frac{l_{\text{j}}}{∥l_{\text{j}}∥}∥r∥\text{cos}\beta \right)\right\}$$ (10)

where

$$\text{cos}\beta =\frac{l_{\text{j}}^{\text{T}}r}{\left\|l_{\text{j}}\right\|\left\|\text{r}\right\|}$$ (11)

And so

$$\Delta l_{\text{j}}=\xi \left\{r-\left(l_{\text{j}}{\left(l_{\text{j}}^{\text{T}}l_{\text{j}}\right)}^{-1}l_{\text{j}}^{\text{T}}r\right)\right\}$$ (12)

The magnitude of Δ**l**_j_ is controlled by the parameter ξ which can be used to manipulate the amount by which the forward field is modified.

The single source simulations, described in **Reconstruction accuracy for single dipoles**, were repeated, but this time, sources were reconstructed using beamforming (Eq 8) with added error on the forward field vector. The elements of **r** were defined as Gaussian-distributed random numbers and ξ was varied through 20 iterations such that ‖Δ**l**_j_‖/‖**l**_j_‖ spanned approximately the range from 0 to 0.5. For each value of ξ in the simulation was repeated 5 times (with 5 realisations of **r**) and results averaged. This whole process was repeated for 100 dipole locations randomly selected from one cortical hemisphere (performing this calculation for all dipole locations proved too computationally intensive). Results were averaged across locations and differentiated by distance to their closest sOPM sensor, d_j_.

### Spatial resolution and channel count

To examine the spatial resolution of source-reconstructed MEG data from the sSQUID and sOPM systems, a final set of simulations was employed. Two sources were simulated at separate locations on the cortical surface, k and n. Two source time courses, **q**_k_ and **q**_n_ were simulated; both contained a total of N time points and comprised different Gaussian-distributed random data with a standard deviation of 5 nAm, meaning that the sources were temporally orthogonal. MEG data were then generated as

$$m\left(\text{t}\right)=l_{\text{k}}q_{\text{k}}+l_{\text{n}}q_{\text{n}}+e$$ (13)

where **l**_k_ and **l**_n_ are the forward field vectors for cortical locations k and n respectively, and **e** represents sensor noise (normally-distributed random data with a standard deviation of 35 fT). This simulation was run multiple times with the source locations (k and n) selected randomly. The Euclidean distance between sources was measured for each source pair.

As previously, data were reconstructed using the beamformer spatial filter such that **q̂**_k_ = **w**_k_^T^**m** and **q̂**_n_ = **w**_n_^T^**m**. In a connectivity measurement in real MEG data, we would look to quantify a relationship between the two dipole time courses, **q̂**_k_ and **q̂**_n_; any statistical interdependency between these quantities would be used to indicate a functional connection between brain regions k and n. However, such a measurement necessarily requires that there is no spurious correlation between **q̂**_k_ and **q̂**_n_ caused by the limited spatial resolution. In the present simulation, the underlying time courses **q**_k_ and **q**_n_ used to generate the data are orthogonal, so that the correlation coefficient r(**q**_k_,**q**_n_) = 0. In order to test for the effects of spurious correlations in the beamformer reconstruction, we therefore measured correlation between source reconstructions, r(**q̂**_k_,**q̂**_n_); if the dipoles simulated at k and n are resolvable, then this quantity should be close to zero. Note that in much of the functional connectivity literature, r(**q̂**_k_,**q̂**_n_) would be referred to as leakage between sources. This simulation was run 3000 times with N = 360,000. The measured Euclidean distances were categorised into 30 different bins of 2 mm width, the smallest being 0–2 mm, the largest being 58–60 mm. The simulation was designed such that 100 repeats were run for each bin. The spatial resolutions of the sSQUID and sOPM systems were then measured via assessment of the quantity r(**q̂**_k_,**q̂**_n_) plotted against distance; two sources were considered resolved if r(**q̂**_k_,**q̂**_n_) < 1/√2 (i.e. less than 50% shared variance between sources).

We also investigated the effect of different channel counts on the sOPM system’s spatial resolution. In order to do this, three additional sensor configurations were simulated according: to 1) the international 10–10 EEG system; 2) the 10–5 EEG system [57] and 3) a theoretical 10–2.5 system. The application of these sensor configurations onto the scalp surface [58] resulted in systems with 81, 329 and 1293 sensors respectively. A separate measurement of r(**q̂**_k_,**q̂**_n_) versus source separation was made for each of these three systems.

Finally, a simulation was employed to quantify the spatial resolution of both systems as a function of cortical location. Simulated MEG data were generated for pairs of dipoles as before (Eq 13), with the only difference that k ran for all dipole locations within one cortical hemisphere and n corresponded initially to k’s nearest neighbour on the cortical mesh. In cases where r(**q̂**_k_,**q̂**_n_) was larger than 1/√2, the simulation was repeated for k’s next nearest neighbour, and so on, until the value of r(**q̂**_k_,**q̂**_n_) fell below 1/√2. Here, spatial resolution was assessed as the minimum distance between the dipole seed (k) and its neighbour (n) for which both sources can be considered separable. Both this spatial resolution value and the correlation between reconstructed time courses was assessed.

## Results

**Fig 3:**
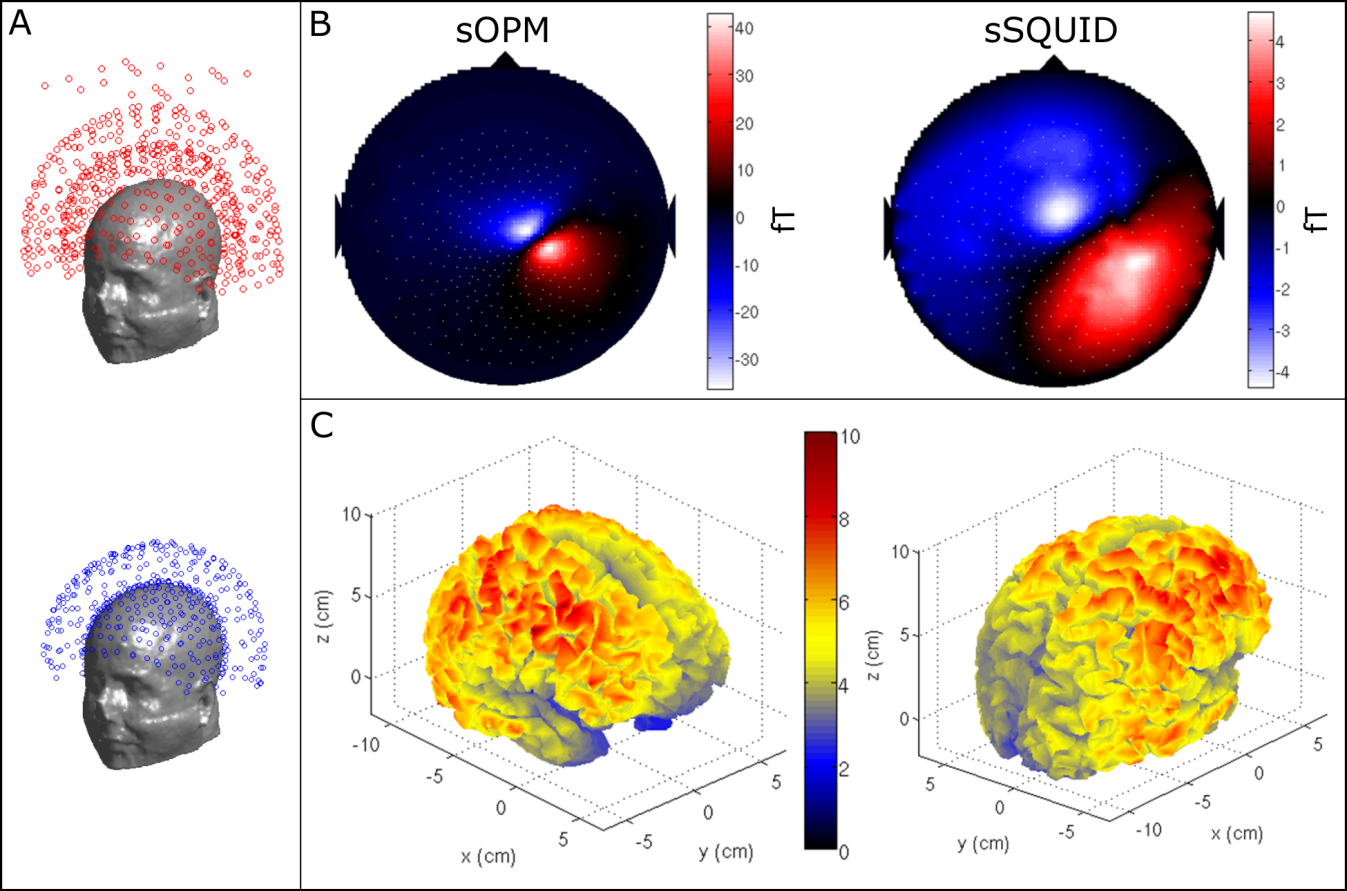
SNR simulation. (A) Simulation set-up; the upper panel shows the coil arrangement for the sSQUID system, based on the axial gradiometer configuration of the 275 channel CTF MEG instrument. The lower panel shows our sOPM system; note 5 cm baseline axial gradiometers are used to allow a direct comparison of the sSQUID and sOPM systems. (B) Simulated magnetic field from a single dipole located in the parietal lobe. Note the increased magnitude and more focal nature of the measured magnetic field patterns. (C) The ratio of the Frobenius norms of the forward fields, plotted as a function of dipole location on the cortex. The colours represent the quantity f_j,OPM_/f_j,SQ_; i.e. a ratio of 5 would indicate a fivefold improvement in SNR of the sOPM compared to the sSQUID system (assuming equal noise floors). The left and right panels show different aspects of the same data.

Fig 3 shows the results of our SNR measurements. Fig 3A shows the basic geometry of our simulations. The upper panel shows the coil arrangement for the sSQUID system which is based upon the axial gradiometer arrangement of a 275-channel CTF MEG instrument. The reference array that is used to form the third-order synthetic gradiometers can also be seen in the figure. The lower panel in Fig 3A shows the sensor arrangement for the sOPM system. Here, again a 5 cm baseline axial gradiometer set-up is employed since this allows a direct comparison of the SNR for the sSQUID and sOPM systems. The sOPM sensor topography across the scalp is based upon the projection of the 275 SQUID channels onto the subject’s scalp. The inner shell of detectors is placed just 4 mm from the scalp surface. Fig 3B shows simulated magnetic fields (i.e. the forward field) for a single dipole located in the parietal lobe. Note that, for this particular dipole, the magnitude of the maximum measured magnetic fields is approximately tenfold higher for the sOPM system than for the sSQUID set-up. As would be expected, the topographic pattern of the magnetic field is also more focal in the sOPM compared to the sSQUID system. Fig 3C shows the ratio of the Frobenius norms of the forward field vectors for the two systems, plotted as a function of dipole location on the cortex. The colours represent the ratio (f_j,OPM_/f_j,SQ_) such that a ratio of 5 would indicate a fivefold improvement in SNR of the sOPM compared to the sSQUID system. The figure shows clearly that the sOPM system offers an improvement in SNR for all dipole locations on the cortex; this improvement is approximately a factor of five (yellow) across most of the cortex, increasing to close to ten (red) for some areas of the frontal lobe. The improvement is less dramatic in deeper cortical areas (a consequence of the inverse square law), but the sOPM system still offers twice the SNR of the sSQUID system (blue). Note that the spatial variation of this improvement in SNR depends on the position of the subject’s head in the CTF MEG helmet. As stated above, in the simulations the subject was positioned with the back of their head touching the back of the CTF-system helmet, thus the largest improvements in SNR are observed in the frontal lobes. Note also that these measurements were based on an adult male, with a head size of 21.3 cm anterior to posterior and 14.6 cm left to right, meaning that this individual’s head was well fitted to the size of the CTF helmet: in a subject with a small head, the improvements provided by the sOPM system would be more pronounced.

**Fig 4:**
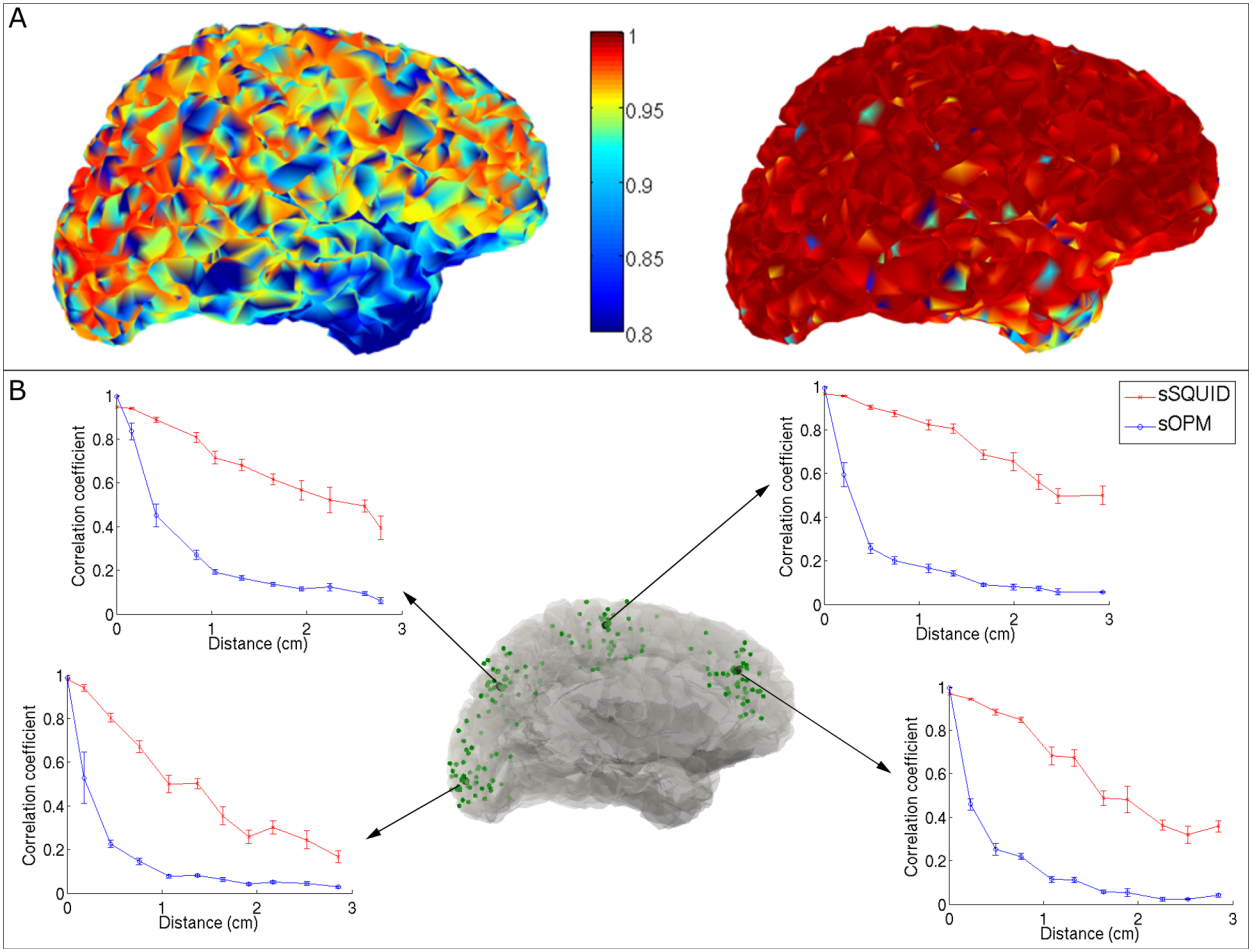
Reconstruction accuracy. (A) The left hand panel shows beamformer reconstruction accuracy for the sSQUID system. The right hand panel shows beamformer reconstruction accuracy for the sOPM system. In both cases reconstruction accuracy is measured as temporal correlation between the simulated and reconstructed dipole time courses. Colours represent the quantities r_SQ_(**q**_j_,**q̂**_j_) and r_OPM_(**q**_j_,**q̂**_j_) for left and right panels, respectively. Note the improvement for the sOPM system. (B) Spatial reconstruction accuracy around four cortical locations (in black). The four inset graphs show correlation coefficients between the original simulated time course and reconstructed dipole time courses at the simulated source location and its nearest neighbours (shown in green on the central image). Correlation is plotted as a function of Euclidean distance to the simulated dipole. Note that for both systems, correlation is maximal closest to the simulated source, however correlation falls off more quickly with distance for the sOPM system, indicating a better spatially resolved image.

Fig 4A shows beamformer reconstruction accuracy in our single dipole simulations (see **Reconstruction accuracy for single dipoles**). The left panel shows the sSQUID system and right panel shows the sOPM system. In both cases the colour bar indicates temporal correlation between the simulated and reconstructed dipole time courses; i.e. the left and right plots show r_SQ_(**q**_j_,**q̂**_j_) and r_OPM_(**q**_j_,**q̂**_j_) respectively, plotted against dipole location j on the cortical surface. A value of 1 would indicate perfect reconstruction. Here, the sSQUID system exhibits reasonably good performance with correlation greater than 0.8 across the vast majority of the cortex. However reconstruction accuracy is highest for the occipital lobe and drops in the frontal lobes—most likely due to the position of the subject’s head in the MEG helmet. Reconstruction accuracy is lowest in the temporal lobes and this is likely due to lower sensor coverage for these brain regions. In contrast, for the sOPM system we see excellent reconstruction accuracy across the entire cortex, with correlation greater than 0.9 everywhere on the cortical surface. Note particularly the improvements in reconstruction accuracy in the temporal lobe compared with the sSQUID system. Fig 4B shows spatial reconstruction accuracy for four different locations (shown overlaid in black on the central figure). Correlation coefficients between the original and reconstructed time courses for the simulated dipole location and 44 of its nearest neighbours, are plotted against the relative distance to the simulated dipole location. Results show that both systems generate an accurate spatial reconstruction of the source with maximum correlation at zero distance. However, temporal correlation falls off faster with distance for the sOPM system indicating a sharper (more focal) reconstruction.

**Fig 5:**
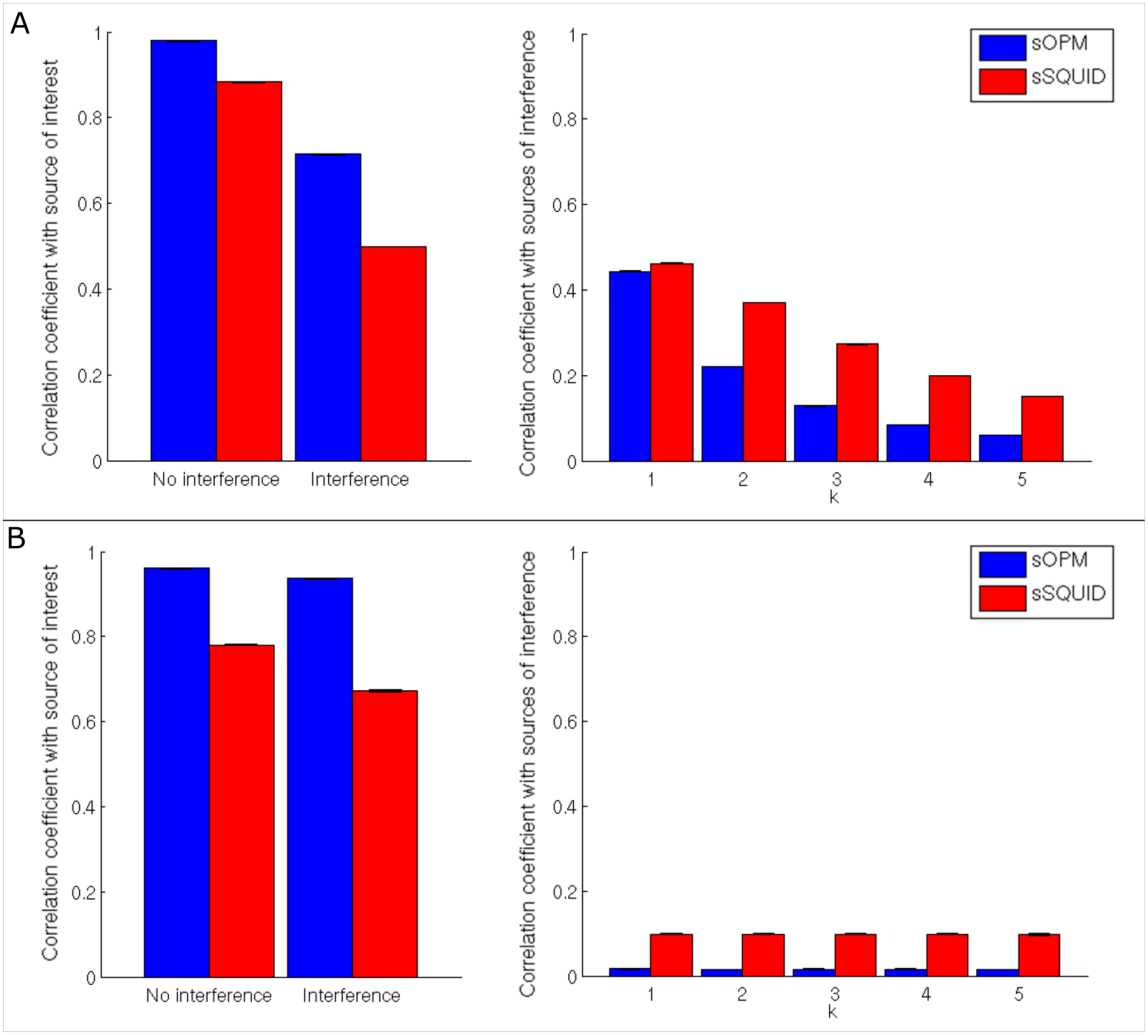
Reconstruction accuracy with “brain noise”. (A) Case 1 (interference in close proximity): left hand panel shows average of correlation coefficients between simulated and reconstructed dipole time courses, r(**q**_j_,**q̂**_j_), with and without interference. Right hand panel shows average of correlation coefficients between each of the interference sources and the reconstructed main dipole time course, i.e. r_Int_(**q**_Int,k_,**q̂**_j_). (B) Case 2 (deep sources): left hand panel represents the average of correlation coefficients between the estimated and the simulated dipole time course, with and without interference sources. Right hand panel shows again average of correlation coefficients with sources of interference.

Fig 5, shows beamformer reconstruction accuracy with “brain noise” (see **Reconstruction accuracy with ‘brain noise’**). Panel 5A shows results for case 1 (interference in close proximity) whilst panel 5B shows results for case 2 (deep sources). In both cases the left hand panel shows average temporal correlation between the simulated and the beamformer-reconstructed source of interest at j (i.e. graphs show the quantity r(**q**_j_,**q̂**_j_)). For comparison, the cases with and without the interference sources are both shown. Right hand plots represent average correlation between each of the 5 simulated interference sources and the beamformer estimate of the source of interest at j (i.e. the quantity r_Int_(**q**_Int,k_,**q̂**_j_)). In Fig 5A (left), it is clear that switching on the interference degrades the quality of the source estimate for both systems, as would be expected; however this degradation is more pronounced for the sSQUID system compared to the sOPM system. Regarding Fig 5A (right), first recall that interference sources were located progressively further away (in terms of Euclidean distance) from the source of interest with increasing index, k; this explains the decreasing temporal correlation with increasing k. Importantly, the beamformer’s ability to reject interference (even in very close proximity) is improved markedly for the sOPM system compared to the SQUID system. Note that the temporal correlation between the beamformer-estimated source of interest and all interfering sources is lower for sOPM than for sSQUID; this is the case for interference in close proximity, and for deep sources.

**Fig 6:**
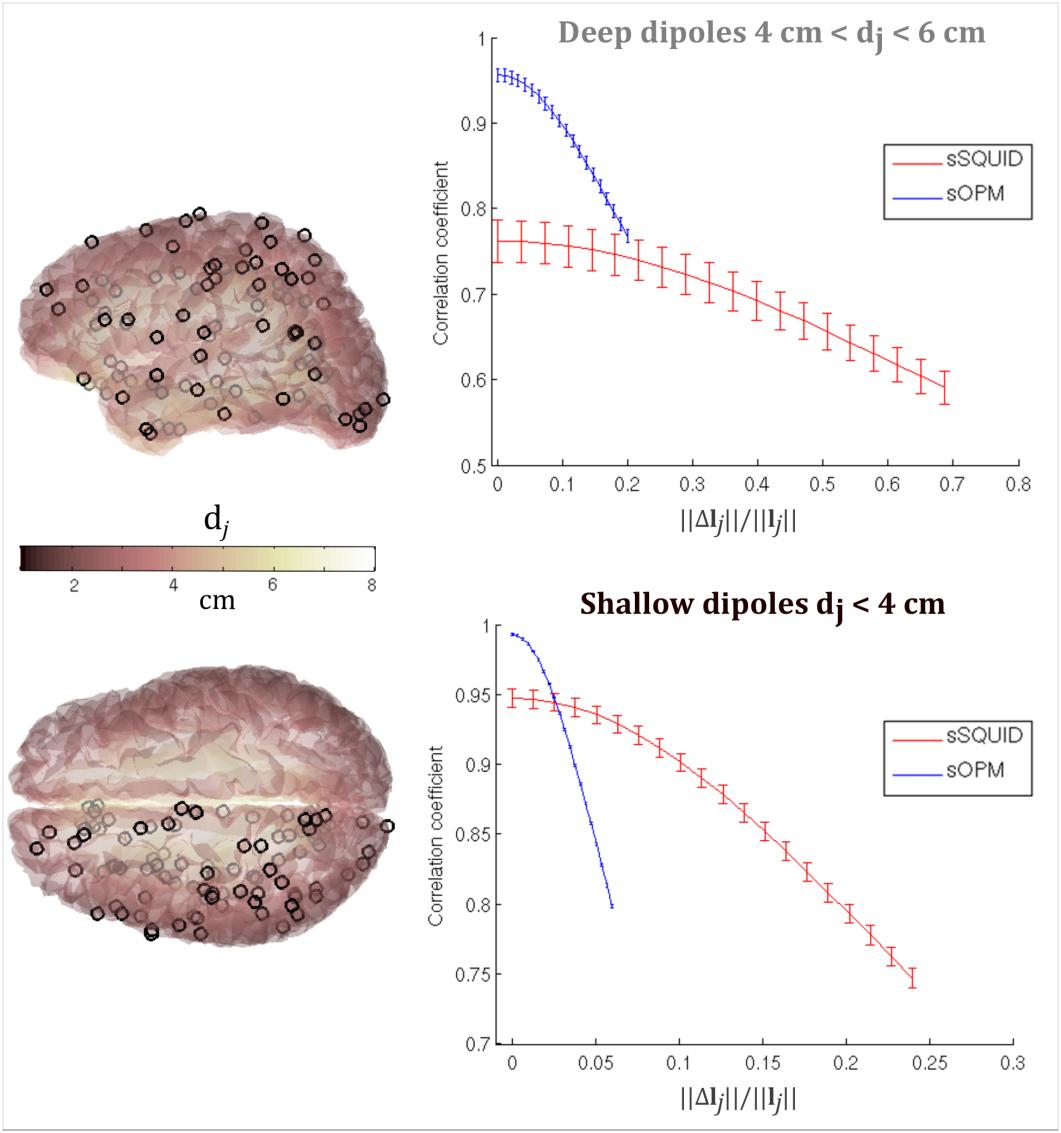
Dependency of reconstruction accuracy on forward field error. Left image shows dipole locations with deep sources shown in grey and shallower sources shown in black. The plots on the right show temporal correlation between the simulated and beamformer reconstructed sources (i.e. r(**q**_j_,**q̂**_j_)) plotted as a function of fractional error on forward field (‖Δ**l**_j_‖/‖**l**_j_‖). The two separate plots show the case for deep (top) and shallow (bottom) dipoles. The sOPM system is shown in blue and the sSQUID system in red. Note that the improvement in reconstruction accuracy afforded by the OPM based system depends on accurate forward field modelling.

Fig 6 shows how reconstruction accuracy is affected by errors in the forward field model (see **Reconstruction accuracy with forward field error**). In order to visualise results, dipole locations on the cortical surface were divided according to depth: those whose distance to the closest sOPM sensor, d_j_, is less than 4 cm (shallow sources—shown in black on the left image) and those whose distance, d_j_, to the closest sOPM sensor is between 4 cm and 6 cm (deep sources—shown in grey on the left image). To show the influence of forward field accuracy on beamforming, correlation coefficients between simulated and reconstructed time courses (r(**q**_j_,**q̂**_j_)) were measured and plotted as a function of fractional error on the forward field ‖Δ**l**_j_‖/‖**l**_j_‖. The top graph shows results for deep sources whilst the bottom graph shows results for shallow sources. In both cases note that, with no forward field error, sOPM outperforms sSQUID. This is in agreement with the results given in Figs 4 and 5. However, as forward field error is increased, the reconstruction accuracy of both systems falls, with the sOPM system being more sensitive. This means that, for dipoles close to the scalp surface (shallow dipoles) even an error of less than 5% in the forward field model can negate the significant advantages afforded by the sOPM system. For deeper dipoles, this is less dramatic, but a 25% error on the forward field would again mean that sSQUID and sOPM systems perform approximately equally well, with errors greater than this value meaning the sSQUID system more accurately reconstructs dipole time courses. These results highlight a critical point; although the introduction of an OPM system has the potential to generate a step change in the utility of MEG, the realisation of this potential depends not only on our ability to engineer such a system, but also critically on our ability to model accurately the forward field. This key point will be addressed further in the discussion below.

**Fig 7:**
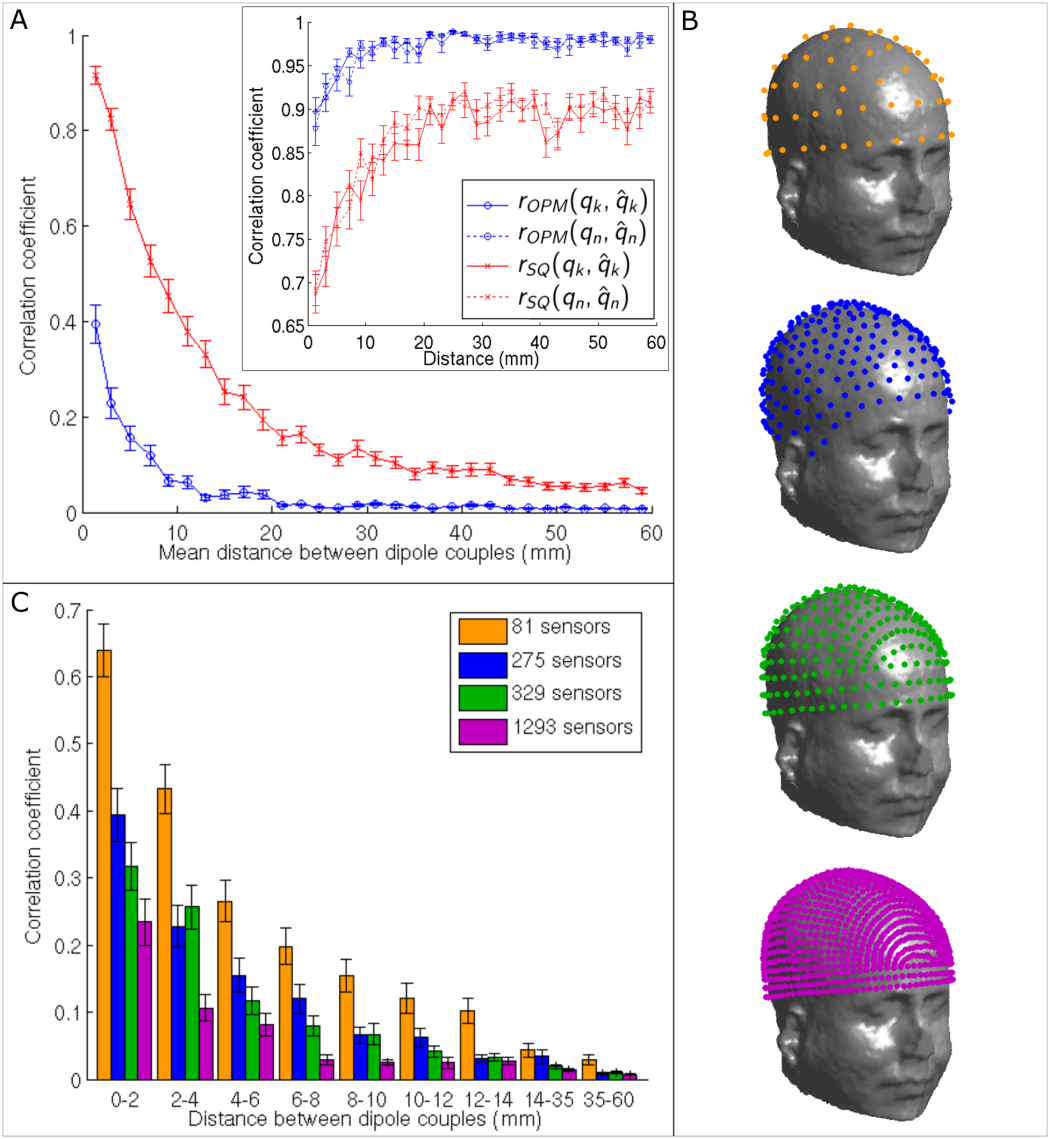
Spatial resolution and channel count measurements. (A) Correlation coefficient between two simulated dipole sources after beamforming reconstruction, r(**q̂**_k_,**q̂**_n_) plotted against Euclidean distance between the sources. The red and blue traces show the results for the sSQUID system and sOPM systems, respectively. Note that the simulated sources were temporally uncorrelated, so a value of zero would indicate perfect reconstruction. Note the improved performance of the sOPM compared to the sSQUID system. The inset shows correlation coefficients between simulated and reconstructed time courses for the two sources separately, i.e. r(**q**_k_,**q̂**_k_) and r(**q**_n_,**q̂**_n_). (B) Simulation set-up comparing four different sOPM systems (from top to bottom): 81 sensors following the 10–10 EEG system; 275 sensors (equivalent to that shown in Fig 1A); 329 sensors following the 10–5 system; 1293 sensors following a hypothetical 10–2.5 system. (C) Comparison of spatial resolution for the 81, 275, 329 and 1293 sensor sOPM systems. Again the graphs show correlation between reconstructed time courses plotted as a function of Euclidean distance between sources. Note the improvement in performance as channel count is increased.

**Fig 8:**
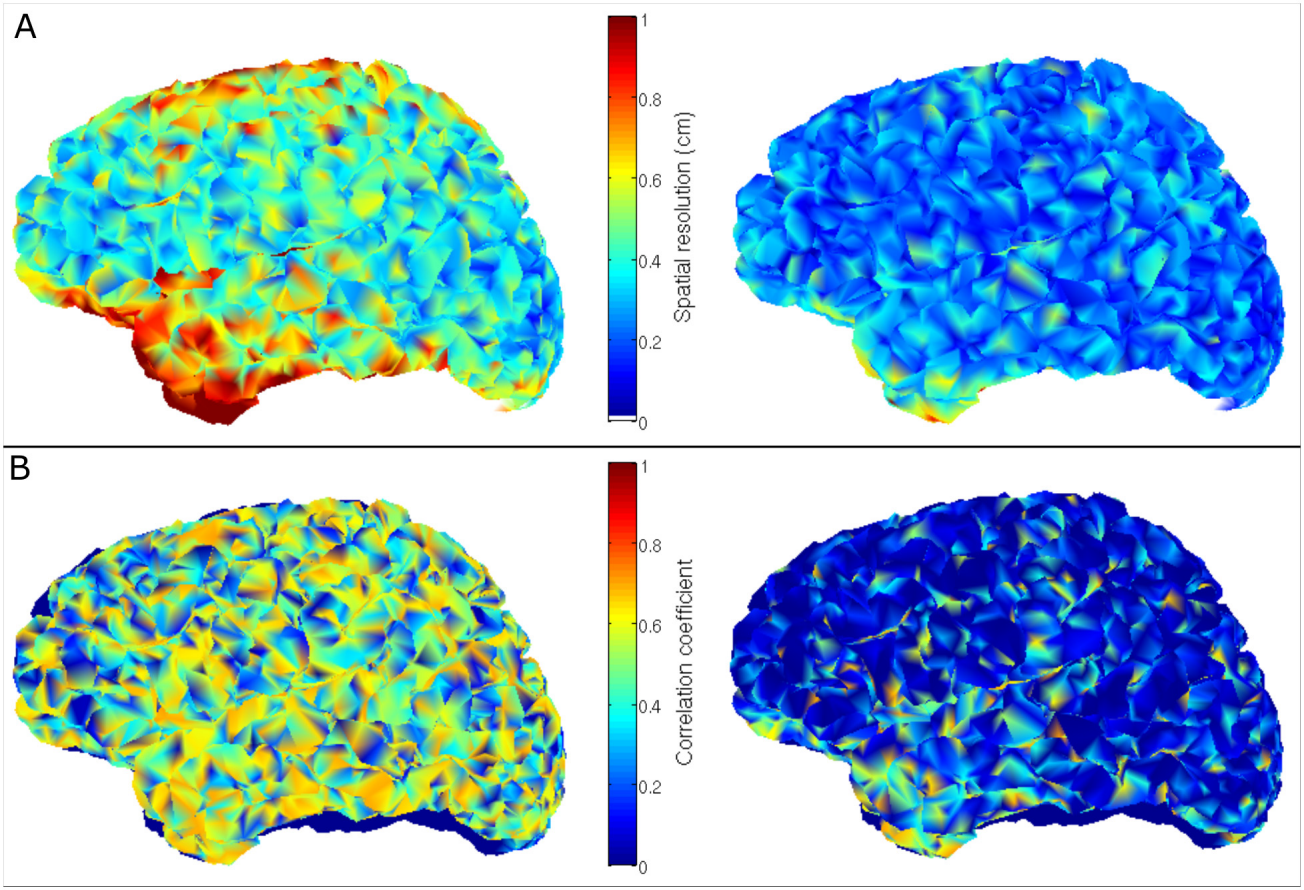
Spatial resolution (275 channels). (A) Minimum distance between the dipole seed and its neighbours for which two sources are separable (defined when temporal correlation between time courses falls below 1/√2) plotted as a function of seed dipole location. Left hand panel shows results for the sSQUID system and right hand panel shows results for the sOPM system. Importantly, the discrete nature of the cortical mesh means that a dipole separation corresponding to a temporal correlation of precisely 1/√2 is not possible. For this reason, the spatial separations in Fig 8A must be given alongside their corresponding temporal correlation values which are shown in (B). The left and right hand panels correspond to sSQUID and sOPM systems respectively. Note smaller spatial separations can be achieved with the sOPM system compared to the sSQUID system (A), especially in the temporal lobe. Note also that in addition to smaller separation, leakage (temporal correlation) is also reduced dramatically (B).

Figs 7 and 8 show the results of our spatial resolution measurements. Fig 7A shows how the correlation, r(**q̂**_k_,**q̂**_n_), between two reconstructed sources (also termed source leakage in connectivity literature—see **Spatial resolution and channel count**) varies with source separation for the sOPM (blue curve) and sSQUID systems (red curve). The simulated source time courses were orthogonal, so any non-zero value of r(**q̂**_k_,**q̂**_n_) is purely an artefact of the limited spatial resolution of the beamformer. Two sources are deemed separable if r(**q̂**_k_,**q̂**_n_) < 1/√2. Note that no forward field error was simulated for results in Fig 7. It is clear that the 275-channel sOPM system offers much improved spatial resolution compared to the sSQUID system. Sources become separable for the sSQUID system when the Euclidean separation is approximately 5 mm. Indeed this value is in approximate agreement with findings from real data [12]. However for the sOPM system, sources are separable at smaller separations, with temporal correlation never reaching more than 0.4 (16% shared variance). This finding shows clearly the significant advantages of an OPM system, particularly in the study of functional connectivity; this will be addressed further in the discussion below. In the inset figure, the correlation coefficients between simulated and reconstructed time courses for each dipole source (r(**q**_k_,**q̂**_k_) and r(**q**_n_,**q̂**_n_)) are also plotted for both systems, as a function of distance between sources. This reflects the reconstruction accuracy for each dipole, showing that each dipole time course is better reconstructed as distance between them is increased. Note again improved performance of the sOPM system. Fig 7B shows four different sOPM systems: (from top to bottom) 81 sensors following the 10–10 EEG system; 275 sensors (as shown in Fig 1A); 329 sensors following the 10–5 system; 1293 sensors following a hypothetical 10–2.5 system. These four simulated systems were used to assess the advantages of increased channel counts for spatial resolution. Fig 7C shows results of this measurement: again r(**q̂**_k_,**q̂**_n_) is plotted as a function of distance between sources for the 81-channel (orange), 275-channel (blue), 329-channel (green) and 1293-channel (purple) OPM systems. A marked improvement in spatial resolution is observed as channel count is increased.

Finally, Fig 8 shows spatial resolution plotted as a function of location on the cortical surface. Fig 8A shows the minimum spatial separation for which two dipoles become resolvable (i.e. the temporal correlation between time courses falls below 1/√2). This in itself does not constitute a measure of spatial resolution because the discrete nature of the cortical mesh means that a dipole separation corresponding to a temporal correlation of precisely 1/√2 is not possible. For this reason, the spatial separations in Fig 8A must be given alongside a corresponding value of temporal correlation at the derived minimum separation. These values are given in Fig 8B. Note that not only is the minimum spatial separation between dipoles reduced from an average of 6.3 mm in the sSQUID system to 2.5 mm in the sOPM system, but also the temporal correlation at those minimum separations is reduced from of 0.49 in the sSQUID to 0.29 in the sOPM. This important result is in agreement with that shown in Fig 7 and further supports the finding that the sOPM system demonstrates dramatically better spatial resolution compared to the sSQUID system.

## Discussion

In this paper, we have used simulations to quantify the potential advantages of an OPM system in terms of SNR, beamformer reconstruction accuracy (with and without interference) and spatial resolution. Our results show that an OPM system, with an equivalent number of channels and equal noise floor to current SQUID systems, would offer approximately a fivefold increase in SNR across most of the cortex, and up to an order of magnitude improvement in SNR in some regions, for an adult brain. This improvement, which results from the closer proximity of the sensors to the cortex, would be even more dramatic for subjects with smaller heads (e.g. infants). Our results also show a clear improvement in the accuracy of beamformer source reconstruction; average correlation between simulated and beamformer-reconstructed sources increased significantly for our simulated OPM system and this was the case with and without interference from other brain sources. However, our simulations also show that such improvement depends critically on the accurate generation of forward field models. Finally, our results show a marked improvement in spatial resolution of the simulated OPM system, which becomes more dramatic when increasing the channel count. Overall, our results imply that the realisation of a multi-channel whole-head OPM system could generate a step change in the utility of MEG as a means to assess electrophysiological brain activity in health and disease. However, such improvements can only result from both improved hardware and improved modelling algorithms.

The SNR improvement afforded by OPM systems would revolutionise both the type of neuromagnetic effects that can be measured by MEG, and the brain regions to which MEG is sensitive. At present, MEG measurements are limited, particularly at high frequency. The sensitivity of the measurement falls with frequency since neural networks oscillating at high frequencies tend to be small, and therefore offer low SNR when magnetic fields are measured at a distance. However, invasive recordings show clearly that neuro-electrical effects up to and including frequencies of 200 Hz are of significant importance to brain function [59]. OPM systems, we believe, could offer the best possible route to assess, non-invasively, networks of neurons oscillating at these high frequencies. The improvement in SNR also offers a better coverage of deep brain areas. This would enhance coverage for deep cortical regions such as cingulate and insula, and also sub-cortical grey matter nuclei including basal ganglia and hippocampi; signals from both of these regions have been measured successfully using conventional SQUID-based MEG systems [26, 60], however efforts to characterise their behaviour have been hampered by low SNR. Our simulations (Fig 5B) show that signals acquired from all deep structures using an OPM system would be enhanced in terms of SNR. However, the degree of improvement is a function of depth. Specifically, the non-linear nature of the inverse square law means that, although moving detectors closer to the scalp surface offers increased SNR for *all* regions, the improvement for shallow sources is proportionally larger than for deep sources. Indeed, Fig 3 shows that whilst improvements for shallow sources could be as high as tenfold, for deeper sources the gain may be closer to twofold. This observation has important consequences. Even in current systems the effective characterisation of deep sources relies on our ability to eliminate interfering signals for shallow sources. When using OPMs, the greater amplification of shallow compared to deep sources makes this problem harder, since the ratio of deep source (of interest) to shallow source (of no interest) signals is markedly reduced. This observation provides more evidence that realising the benefits of an OPM system depends critically on accurate source modelling.

Our simulations also showed that, as a result of improved SNR (Fig 3C) and the more focal nature of the forward field (Fig 3B), beamformer reconstruction accuracy was improved significantly when using OPMs compared to a SQUID-based system. Specifically, temporal correlation between simulated and beamformer-reconstructed cortical sources increased significantly (p < 0.05) from 0.87 for a current SQUID-based system, to 0.98 for a 275-channel OPM system (single dipole model). Improvements were also observed in the presence of interference fields from nearby cortical dipoles of no interest and our results in Fig 5 show that the beamformer algorithm, applied to our sOPM system, was better able to cancel out such interference compared to the sSQUID system.

Although beamformer performance is enhanced in an OPM system, our results in Fig 6 show clearly that such improvements would rely heavily on accurate forward modelling. In the case of shallow cortical dipoles, simulations showed that a forward field error of as little as 4–10% would negate the significant advantages afforded by OPM instrumentation. This effect was also seen for deeper dipoles although it is somewhat less pronounced, with OPMs still outperforming SQUIDs up to approximately a 25% error on the forward field. Whilst perhaps counter-intuitive, this observation is well known in beamforming; in short, as SNR increases, inverse modelling becomes increasingly sensitive to the accuracy of the forward model. In the case of OPMs, the increase in SNR afforded by the shift in sensor locations towards the scalp surface drives this necessity for increased forward field accuracy. In the simulations presented, we use a simple mathematical method in order to artificially generate an error on the forward solution. This simulated error is an abstract mathematical formulation, but nevertheless illustrates the problem. Experimentally, these forward field errors come from 1) inaccurate co-registration between the head location and the sensor geometry, 2) subject movement (the forward field is derived based on average head position) and 3) inaccuracy in the forward model itself (e.g. deviation from the dipole model). In recent years, significant improvements in the latter have been introduced via, for example, the use of boundary element methods (BEM) based upon high resolution MR images. However, the co-registration problem is still heavily reliant on techniques such as surface digitisation and matching; which often leads to significant systematic errors in estimating MEG sensor locations relative to the brain anatomy. In fact, previous work suggests that the largest source of forward modelling error in MEG comes from co-registration [61]. Also, for OPMs, as a consequence of subject movement we might expect independent errors if the sensors are not mounted rigidly with respect to one another. In addition, the OPM’s aperture (sensitive volume to magnetic field) will have a smaller area than that of the SQUID coils, and also have a variable gain (in terms of magnitude and directional sensitivity) which will depend not only on fabrication but also on the proximity (and cross-talk) of other sensors. These errors could also impact significantly on forward field accuracy. Here we demonstrate that an abstract modelling error causes a significant problem that could easily negate any advantages afforded by an OPM system, particularly in modelling shallow cortical dipoles. Future work should include accurate characterisation (in terms of systematic, independent, gain and orientation errors) given a specific OPM sensor type. It is noteworthy that a potential solution to co-registration and subject movement problems is the use of 3D-printed head casts which have been demonstrated in recent work [62] and their use in current helmet-based (SQUID) systems facilitates accurate placement of the head relative to the scanner. If OPM-based systems could be fabricated, with bespoke head casts to hold the individual sensors in accurately known locations relative to the subject’s anatomical MRI, this might represent one way to ensure that forward modelling is not impacted by systematic error in sensor locations. This methodology, coupled with accurate (e.g. BEM) models, will likely allow forward fields to be derived with a sufficient accuracy to realise the potential of OPM-based instrumentation. However this may come at the expense of subject comfort.

Assuming that solutions to the forward field problem can be found, our spatial resolution measurements in Figs 7 and 8 show the clear advantages of an OPM system in separating dipoles in close proximity in the brain. This is important for all studies, but would be particularly beneficial in the study of functional connectivity between brain regions. The study of connectivity (functional and effective) has caused a paradigm shift in the way in which neuroimaging experiments are conducted and, particularly given the recent advances in dynamic connectivity (temporal changes in connectivity between regions over time) MEG now offers the most attractive means to characterise the way in which the brain forms and dissolves a hierarchy of electrophysiological networks of communication, in order to support current processing demand [21, 22]. Although new MEG methodologies allow for unique insights into such network formation, at present, functional connectivity can only be measured reliably via the use of techniques for post-hoc correction of source leakage between brain regions. This source leakage is a direct result of the limited spatial resolution of the MEG beamformer, and multiple techniques including the use of the imaginary part of the coherence [30], the phase-lag index [32] or linear regression [20, 63] must be employed. Whilst these techniques work, they all eliminate genuine zero-phase lag correlation between electrical signals from separate regions. Invasive measures show clearly that such zero-phase lag effects exist in the brain [64, 65] and are currently missed by MEG. Further, MEG is currently also blind to functional connectivity at high frequency due to poor SNR (see also above) meaning that important electrophysiological effects (for example those observed invasively [66]) cannot be probed. Our results imply that post-hoc correction methods would not be required for OPM systems, for the typical networks observed and high frequency connectivity could be measured. This would prove of key importance in future characterisation of brain network behaviour and dynamics.

It is important to point out the limitations of the simulations that have been undertaken. First, these simulations are simple: they are based on small numbers of dipoles. Second, the noise is assumed to be Gaussian-random and therefore uncorrelated across MEG channels. Although these assumptions are unrealistic compared to experimental measurement, it is worth noting that our source reconstruction results are approximately in agreement with what is observed in real data from current SQUID-based systems [12]. Furthermore, simple simulations of this type have been used extensively in a wide variety of published research [54, 67] and these papers provided inspiration to the field of MEG source reconstruction for more than a decade. The quantitative observations made are therefore likely to be representative, and provide a benchmark to which any real system might aspire. There are also many aspects of OPM system design which we have not touched upon. Most importantly, OPM sensors offer a means to measure the magnetic field in two orthogonal orientations perpendicular to the laser beam. This means that in addition to measuring magnetic field in the radial direction (the approach taken by all conventional systems), one additional tangential direction may also be measured using the same sensor. Practically, this requires extra instrumentation (an extra field modulation coil on each sensor is required, as well as an increased number of readout channels). From a modelling point of view, the tangential component of magnetic field is mainly affected by volume currents and so its measurement would necessitate the use of more complex finite element forward models. Third, from a system design point of view, it is not immediately obvious how one might orient the additional axis (i.e. at each measurement location, which tangential field component should be measured). This latter point in particular is the reason that, in the present paper, we limited ourselves to assessing the radial field only. In fact, design of a system which measure vector field components would necessitate significant simulations to identify an optimal sensor arrangement. However, future simulation work should begin to assess this potentially significant advantage of OPMs compared to SQUIDs. There are other significant design problems with OPMs; for example they operate around a zero field resonance meaning that compensation coils are often mounted on the OPM itself to cancel stray Earth’s field. In addition, the operation of a multi-channel array may be complicated by cross-talk between sensors from both the internal field zeroing coils and from field modulation used for lock-in detection. Interfering fields between sensors in close proximity would significantly alter both the sensor gain and potentially the orientation of the sensitive axis of detection. Our paper does not claim to solve these practical issues relating to the practical use of OPMs, rather it was our intention to present a vision of the potential advantages of an OPM system as well as to elucidate the potential modelling problems that the realisation of such a system might generate. Finally, note that, in principle, the theoretical systems described here could result from any technology in which the external surface of the magnetic field sensor is at room temperature. However, recent demonstrations of micro-fabricated, OPM sensors suggest that this technology represents the basis of viable MEG hardware.

Ultimately, the potential for OPM sensors to replace SQUIDs as the fundamental building block of MEG devices depends upon more than simple arguments relating to SNR. OPMs have the potential to become relatively inexpensive to construct. Furthermore, because OPMs operate free from cryogenic cooling, there is no reliance on liquid helium which is both costly and in short supply. These factors combined make OPM-MEG potentially cheaper in terms of both installation and running costs compared to current machines. More importantly, the flexibility to place sensors anywhere on the scalp surface not only increases SNR, but further increases the patient populations that can be studied, and the types of paradigms that can be employed. The most obvious potential benefit of this flexibility is in the imaging of infants; however, this flexible sensor placement will also allow better coverage of different regions, for example allowing measurement of electrical activity in the cerebellum, or even the spinal cord. Further, OPM array geometry can be optimised via modelling to specify bespoke sensor array patterns to target specific structures. Such flexible determination of the sensor array is a significant consideration in the argument to replace SQUIDs with OPMs. These arguments coupled with the observations made here suggest that OPMs have a bright future in in the development of a new generation of MEG hardware.

## Conclusion

MEG exhibits great potential as a tool for generating a new understanding of human brain function. However, it is limited by low SNR which is caused by the inherently small magnetic fields generated by the brain and by the distance from the scalp surface to the MEG sensors. In recent years, OPMs have become a viable alternative to superconducting devices for MEG, bringing the advantage that they can be brought to within ~4 mm of the scalp surface, thus offering increased SNR. Here we have quantified the advantages of an OPM system in terms of SNR, beamformer reconstruction accuracy and spatial resolution. Our results show that such an OPM system could offer up to an order of magnitude improvement in SNR for an adult brain. Further, our results show clear improvement in reconstruction accuracy and a marked improvement in spatial resolution. Our results imply that the realisation of a multi-channel whole-head OPM system could generate a step change in the utility of MEG as a means to assess human electrophysiological brain activity and connectivity in health and disease. However such a change is critically dependent, not only on the generation of new multi-channel OPM systems, but also on improved algorithms modelling for MEG data.

## Supporting Information

S1 Data File containing the data used to replicate the figures. (ZIP)

S1 Supporting Information Description of the contents in S1_data.zip file. (PDF)
