# Supplementary material for: On the Potential of a New Generation of Magnetometers for MEG: A Beamformer Simulation Study
Source: PLoS One. 2016 Aug 26;11(8):e0157655. doi: 10.1371/journal.pone.0157655 (PMC5001648; doi:10.1371/journal.pone.0157655)
Supplement: S1 Supporting Information — (PDF) [file pone.0157655.s002.pdf]

Supporting Information

SupportingInformation.zip file contains all the data used for this paper.
All units of distances are expressed in centimetres.

Meshes and sensors

- mesh.mat contains faces and vertices of the surfaces:
  - Cortex
    - Faces: (42,794x3)
    - Vertices: x, y and z coordinates (21,401x3)
  - Head
    - Faces: (23012x3)
    - Vertices: (35,167x3)
- sensors.mat contains position (Nx3) and orientation (Nx3) of the different channel systems (being N the number of channels).
  - sSQUID: N = 270 (CTF system, gradiometers)
  - sOPM:
    - 275: N = 270 (gradiometers and magnetometers)
    - 10-10: N = 81 (gradiometers and magnetometers)
    - 10-5: N = 329 (gradiometers and magnetometers)
    - 10-2.5: N = 1293 (magnetometers)

SNR

- Lead fields: (270x21,401) sSQUID and sOPM
- Frobenius norms: (1x21,401) sSQUID and sOPM
- Ratio: (1x21,401)

Reconstruction Accuracy

Single dipoles

- Temporal correlations: (21,401x2) correlation coefficients between simulated and estimated dipole time courses (first column: $r_{SQ}(q_j,\hat{q}_j)$, second column: $r_{OPM}(q_j,\hat{q}_j)$).
- Spatial performance:
  - Seeds: (4x1) cortical nodes' indices of seeds.
  - Neighbours: (4x50) cortical nodes' indices of neighbours.
  - Distances: (4x51) distances between seeds and corresponding neighbours.
  - Correlations: (4x51) correlation coefficients between seed's simulated time course and neighbours' reconstructed time courses ($r(q_j,\hat{q}_k)$). sOPM and sSQUID.

Brain noise

- Close proximity
- Deep sources

In both:

- Seeds' and neighbours' cortical indices.
- Correlation coefficients between:
  - Seed's simulated and estimated time courses ($r(q_j,\hat{q}_j)$, first column: without interference, second column: with interference).
  - Seed's estimated and neighbour's simulated time courses ($r(q_k,\hat{q}_j)$, each column corresponds to each interference source, k = 1, 2, …, 5).

Forward field error

- Seeds: (100x1) cortical nodes' indices of deep (1 to 50) and shallow (51 to 100) sources.
- Fractional error: (100x20) error on forward field vectors.
- Correlations: (100x20) correlation coefficients between simulated and estimated time courses ($r(q_j,\hat{q}_j)$) of deep and shallow dipoles. Columns correspond to increasing error values on the forward field.

Spatial resolution

sSQUID and sOPM275

In both:

- Distances between dipole pairs (3000x1) ranging from 0 to 6 cm, in 2 mm bins, i.e. 100 dipole pairs in each bin (30 bins in total).
- Correlation coefficients:
  - Between simulated and estimated time courses, (3000x2). First column: $r(q_k,\hat{q}_k)$, second column: $r(q_n,\hat{q}_n)$.
  - Between estimated time courses, $r(\hat{q}_k,\hat{q}_n)$ (3000x1).

sOPM 10-2.5, 10-5 and 10-10

- Distances between dipole pairs (900x1), first 700 dipole pairs within 0 to 1.4 cm (100 in each 2 mm bin), and the rest 200 within 1.4 and 6 cm (100 within 1.4-3.5 cm and 100 within 3.5-6 cm).
- Correlation coefficients between estimated time courses (900x2) (first column: $r_{SQ}(\hat{q}_k,\hat{q}_n)$, second column: $r_{OPM}(\hat{q}_k,\hat{q}_n)$).

Half cortex

- Correlation coefficients between seed's estimated and neighbour's estimated time courses (12,000x2) (first column: $r_{SQ}(\hat{q}_k,\hat{q}_n)$, second column: $r_{OPM}(\hat{q}_k,\hat{q}_n)$).
- Spatial resolution in centimetres (12,000x2). First column: sSQUID, second: sOPM.
